# Environmental Performance of the Tourism Sector from a Gender Diversity Perspective

**DOI:** 10.3390/ijerph18168834

**Published:** 2021-08-22

**Authors:** Yakira Fernández-Torres, Milagros Gutiérrez-Fernández, Clara Gallego-Sosa

**Affiliations:** 1 Department of Financial Economics and Accounting, Faculty of Business, Finance and Tourism, University of Extremadura, 10071 Cáceres, Spain; yakiraft@unex.es (Y.F.-T.); mgutierrezf@unex.es (M.G.-F.); 2 Department of Economics, Faculty of Economics and Business, University of Extremadura, 06006 Badajoz, Spain

**Keywords:** Sustainable Development Goals (SDGs), environmental performance, gender diversity, board of directors, tourism sector, critical mass

## Abstract

The tourism sector is a driver of economic development characterised by its environmental impact. It is a prevalent part of the 2030 Agenda, given its potential to help meet the Sustainable Development Goals (SDGs). At the same time, board gender diversity is considered essential for companies to implement environmentally sustainable initiatives. However, analysis of the relationship between the role of women on boards and environmental performance has been neglected in the tourism literature. This paper adopts a novel approach to the study of this sector by analysing the relationship between gender diversity on the board of directors and companies’ environmental practices. A fixed effects model is estimated using an international sample of 120 listed tourism companies for the period 2002 to 2019. The results show that boards that are more gender diverse and have a greater female presence are associated with poorer environmental performance and a weaker implementation of policies and practices to reduce resource use and emissions. However, board gender diversity aids performance in environmental innovation.

## 1. Introduction

In September 2015, the United Nations Assembly approved the 2030 Agenda, defined as a ‘plan of action for people, planet and prosperity’ [1] (p. 1). Its aim is to contribute to the sustainable development of the planet by targeting 17 major challenges, formalised as the Sustainable Development Goals (SDGs). Most fall within one of the three dimensions of corporate social responsibility (CSR): the economic, the social and the environmental. Achieving these goals requires effective joint action by governments, civil society and the private sector [1]. Focusing on the role of business, tourism plays a relevant role in the 2030 Agenda. Notably, it is dealt with in 3 of the 17 SDGs: SDG 8 (Decent Work and Economic Growth), SDG 12 (Responsible Consumption and Production) and SDG 14 (Life Below Water). These SDGs are directly linked to the tourism sector because of this sector’s capacity to contribute actively to meeting these goals. From an economic perspective (such as SDG 8), tourism is a key sector for socio-economic progress given its contribution to employment [2,3], the economy, international trade and infrastructures [4,5]. However, the activity of tourism companies also raises considerable challenges that must be addressed (see SDGs 12 and 14). Several studies have reported the adverse effects of tourism on the environment in the form of, for example, greenhouse gas (GHG) emissions, which cause climate change [6,7,8]. It is also estimated that the rapid growth of the sector may double the use of natural resources from 2010 to 2050 [9].

Given the interrelationships among the SDGs, tourism activity indirectly affects other challenges in addition to those mentioned earlier. For example, according to Borja et al. [10], the environmental impact of tourism will have repercussions for public well-being and health, an issue covered by SDG 3 (Good Health and Well-being). The negative role of tourism in this sense stems from the fact that environmental pollution causes cancer [11], respiratory problems and cardiovascular disease [12] and affects the nervous system, increasing stress [13], among other effects. Consequently, the relationship between tourism and climate change has become an intensely debated issue in recent times [8].

From a social perspective, the potential of the tourism sector to create employment [3] means that it can contribute to meeting SDG 5 (Gender Equality), which aims to achieve gender equality and empower women and girls [1] through decent work opportunities for women [14]. At the management level, although the glass ceiling that prevents women from climbing the corporate ladder has weakened in recent decades [15] and a higher proportion of women now occupy management positions [16], women still face difficulties in their career development in the tourism sector. This situation highlights the need to address this issue.

Linked to the previous discussion, there is a close relationship between gender diversity in the tourism sector and the environment. Adopting a gender focus to understand the differentiating impact of tourism in terms of sustainability is crucial [17]. Changes in the environmental sustainability of companies can be explained by the potential influence of women board members in this area [18]. Therefore, achieving gender diversity on the board of directors is essential for companies to undertake environmental actions [19].

However, despite the extensive literature on tourism in recent decades, research on gender diversity in this sector and its consequences remains a minor topic [20,21]. Moreover, studies of tourism and climate change from a gender perspective are scarce [22], highlighting the importance of research in this area [23]. Specifically, only one study seems to have examined the relationship between gender diversity and CSR in terms of each of its three dimensions (economic, social and environmental) in tourism companies [24]. The study showed a positive influence of board gender diversity on environmental performance. However, the literature on the role of gender diversity in environmental performance includes studies of other sectors, although these studies reveal a lack of consensus in their findings. While numerous studies suggest the positive impact of actions by women directors in this area [25,26,27], others indicate that board gender diversity is not a differentiating factor in terms of companies’ environmental performance [28,29]. Some studies even suggest that organisations with higher female representation on their boards have inferior environmental performance [30,31].

To summarise, the discussion so far highlights the importance of the tourism sector as a driver of economic development and a potential agent of change in the fight against climate change, the key role of gender diversity in environmental sustainability and the virtually non-existent evidence of this relationship in tourism companies. These are the principal motivations for this research. The aim of this study is to analyse the influence of tourism companies’ board gender diversity on these firms’ environmental practices. A fixed effects model was estimated using an international sample of 120 listed tourism companies for the period 2002 to 2019.

This research makes several contributions to the literature. First, this study offers what seems to be the first ever analysis of the influence of board gender diversity on environmental performance that focuses exclusively on the tourism sector. A further novel feature of the study is the use of not only a general environmental performance indicator as the dependent variable but also indicators for each of the three dimensions of environmental performance: reduction of resource use, reduction of emissions and innovation. Given the distinctive characteristics of this sector and its strategic role in the fight against climate change, evidence of the factors that condition corporate governance and the consequences for firms’ environmental performance is valuable. This study thus confirms the importance of considering gender-related issues when explaining initiatives by tourism companies to help care for the planet. The study also shows the importance of reaching a critical mass of women on the board of directors in order for their presence to influence environmental policy. This theory has been virtually ignored in studies of gender diversity and environmental sustainability [32]. Finally, the inclusion of a larger number of gender variables than is usual in the literature makes the study novel and relevant.

This paper is divided into six sections, the first being this introduction. Next, Section 2 offers a literature review, summarising the existing theories that support the relationship of interest, as well as evidence of the role of gender diversity in corporate environmental performance. Section 3 describes the sample selection and choice of variables. Section 4 outlines the method. Section 5 presents and discusses the results. Finally, Section 6 presents the conclusions, limitations and possible future lines of research.

## 2. Theoretical Framework

### 2.1. Theories of the Role of Women on the Board of Directors and the Influence of Women on CSR

The relationship between the presence of women on the board of directors and CSR performance is supported by several theories. According to these theories, gender diversity contributes to the effectiveness of boards of directors. This position is supported by various arguments used to provide a theoretical foundation in the literature [33,34,35]. Hence, these theories, which are described below, are used as the basis for this paper.

The first, resource dependence theory [36], is based on the notion that companies act within an open system, making them dependent on their environment. In line with this idea, the board of directors is an important part of corporate performance because it plays the role of intermediary between organisations and their context. From this viewpoint, the board performs two essential functions. First, by opening information channels between the company and its environment, it reduces the uncertainty under which the company operates. Second, the board provides critical organisational resources such as experience and knowledge [37,38]. Through these two functions, gender diversity plays an essential role in company performance for several reasons. Specifically, having women on the board provides new ways to access resources and information [39], greater cognitive diversity [40], different points of view and greater policy options [41]. These outcomes of gender diversity result in more effective decision making [33,42], which leads to better board performance in a variety of areas, including CSR [43]. In addition, women have greater awareness and experience in social and environmental issues [34], further supporting the arguments that underpin the results of studies based on resource dependence theory. These results indicate that companies with higher female representation on their boards have better CSR practices and environmental performance [26,44].

The second theory, stakeholder theory [45], presents the idea that a company cannot focus solely on benefiting shareholders. Instead, it must address the interests of other stakeholders that are also affected by its actions and can in turn affect its activity. Examples include employees, society and the environment. From this perspective, women have been found to have a stronger social orientation than men [46]. Together with a more inclusive personality, this orientation leads to greater concern for stakeholders and attention to their needs [47], resulting in a greater number of social and environmental practices to meet stakeholder expectations [34,48]. Several studies support this theory in terms of the positive effect of gender diversity on environmental performance [49,50,51].

These organisational theories are complemented by social role theory [52], which is based on the idea that behaviour differs between men and women because it is determined by expectations according to the gender roles within a given society [52,53]. For example, the female gender is associated with the role of caring for children and housework. This role means that, in general, women are more understanding and attentive than men [54]. Women are also considered more collaborative [55], democratic, participative and interactive [33], which enables them to communicate more effectively with their subordinates [56] and give a voice to minority groups, aiding their empowerment [43]. In addition, women are more transparent because they advocate greater disclosure of practices, such as social responsibility practices [57,58]. These characteristics of the female gender explain why leadership styles differ between men and women. Accordingly, the effect of corporate governance on company performance is conditioned by gender diversity among directors [19].

In relation to these arguments, critical mass theory also refers to the number of women on the board of directors [59]. Under this theory, the inclusion of women on the board may be a merely symbolic gesture to comply with regulations and improve an organisation’s image [60]. Thus, the female gender, which is considered a token, would not improve the effectiveness of the board of directors [61]. Consequently, it would not influence the company’s CSR practices either [62]. Based on these arguments, the concept of tokenism means that women can only provide new points of view and improve a group’s actions, such as by increasing social and environmental responsibility, if there is a minimum representation of women in that group, known as a critical mass [63,64,65]. Kanter [59] defined four types of groups depending on the percentage of minority members: uniform (absence of minority members), skewed (less than 20% minority members), tilted (20% to 40% minority members) and balanced (40% to 60% minority members). According to the classification proposed by Kanter [59] it is essential to achieve a “tilted” group by having at least 30% women on the board of directors [66], which is equivalent in absolute terms to three women [67], to bring about significant, perceptible changes in group dynamics [68]. Based on these arguments, several studies have confirmed the need for representation of at least three women on the board in order for their presence to contribute positively to socially responsible actions [69] such as environmental initiatives and programmes [70]. However, some scholars disagree, reporting that reaching a critical mass of two women is sufficient to reduce GHG emissions [71] and ensure greater environmental innovation [72].

This section addresses the main theories underpinning the relationship of interest in this research. The next section discusses the evidence of the role of gender diversity in corporate environmental performance.

### 2.2. Board Gender Diversity and Environmental Performance

Multiple studies have shown that board gender diversity plays an essential role in a company’s environmental performance [50,73]. These studies are based on differences in behaviour between men and women. Specifically, women are more aware of the exploitation of the planet [74], more sensitive to environmental problems and more likely to tackle the needs of stakeholders [43,75,76]. Therefore, the presence of women on the board shifts the orientation of companies towards tackling environmental sustainability [18] and meeting SDG 13 (Climate Action) [77]. It also contributes to the implementation of environmental policies and initiatives [78,79,80] and green entrepreneurship programmes [81], all of which results in improved environmental performance [82].

However, the literature is far from offering a consensus on the positive influence of gender diversity on environmental performance. This lack of consensus is reflected in Table 1, which summarises the empirical evidence reported in previous studies. Table 1 shows which type of environmental performance variable was used (a general indicator or a specific indicator for a given environmental dimension). The results for the relationship of interest are also shown.

Assuming that the leader’s gender determines performance in terms of corporate practices, Table 1 shows a number of studies in which board gender diversity is identified as central for enhancing environmental performance (positive relationship). This relationship has been confirmed for samples from various countries and regions. Specifically, studies have been conducted in the United States [26,42,43,50,64,83], France [47], the United Kingdom [25], China [86], Australia [85], Europe [27,77,88] and globally [44,73,96].

Furthermore, the positive influence of board gender diversity on environmental performance has also been corroborated for various sectors, even though sector is a conditioning factor for the sign and significance of the causal relationship between gender and environmental performance [30]. Specific sectors where such research has been conducted include tourism [24], banking [32], energy [51], oil and gas [82] and manufacturing [106]. In the most polluting sectors, the presence of female board members has a more pronounced effect on the adoption of environmental policies [26,79] and environmental innovation [72,91,92].

Besides showing that greater inclusion of women on the board positively influences environmental performance in general, some studies have focused on specific aspects of the environment. For example, Biswas et al. [85] found that companies with greater board gender diversity implement policies and take actions related to reducing resource use and emissions, as well as environmental product and service innovation. Similarly, Orazalin and Baydauletov [27] and Burkhardt et al. [47] have corroborated the positive influence of female representation on the reduction of resource use. The contribution of women directors to emissions reduction policies [27] and environmental innovation [47] has also been reported.

Several authors also claim that although the presence of women directors leads to the implementation of social practices, the same cannot be said of environmental performance. For instance, Cuadrado-Ballesteros et al. [104] and Beji et al. [105] have suggested that although board gender diversity positively influences CSR practices, it does not positively influence the environmental dimension of CSR. Alazzani et al. [103] reported that while board gender diversity gives rise to improved social action by firms, the same is not true in relation to the environment. Such findings have been corroborated by studies that exclusively examine environmental performance [97,106].

Focusing on specific issues, female representation on the board does not influence the company’s approach to climate change [101]. Moreover, it does not lead to greater environmental awareness [100] or determine energy use [29]. This lack of female influence on corporate action may owe to the under-representation of women on the board [28,95,97] or to resistance that reduces women’s voice in decision making because of the stereotype of men as the dominant gender [107].

Finally, some studies have shown a negative relationship between these two variables. However, there are considerably fewer than those showing a positive or neutral relationship, as reflected by Table 1. For example, Reyes-Bastidas and Briano-Turrent [93] reported that while the presence of women on the board favours performance in terms of the economic dimension of CSR, it negatively influences performance in terms of the social and environmental dimensions. Deschênes et al. [30] reported that companies with greater female representation on the board have better CSR practices, but not in the environmental sphere.

Ardito et al. [31] and Shu and Chiang [94] also found that as the number of women on the board of directors increases, environmental performance worsens. These results can be justified by two lines of argument. First, women may be welcomed onto the board of directors merely to improve the company’s image, without any real interest in environmentally friendly practices [30]. Second, communication problems and interpersonal conflicts among board members may emerge due to greater diversity [108]. These issues can result in poorer decision making because it becomes more difficult to reach a consensus on critical decisions [109].

In conclusion, although some studies suggest the absence of a relationship between board gender diversity and corporate environmental performance, or even that this relationship is negative, the bulk of the literature provides evidence of the positive role of women directors in improving corporate environmental performance. Following this reasoning and the argument that women show greater concern for the environment and tend to take actions that are favourable, or at least less harmful, to the planet [110], the following research hypotheses are proposed:

**Hypothesis** **1** **(H1).** *The presence of women on the board contributes to better environmental performance in the tourism sector.*

**Hypothesis** **2** **(H2).** *The presence of women on the board contributes to better environmental performance by reducing the use of resources in the tourism sector.*

**Hypothesis** **3** **(H3).** *The presence of women on the board contributes to better environmental performance by reducing emissions in the tourism sector.*

**Hypothesis** **4** **(H4).** *The presence of women on the board contributes to better performance in terms of environmental innovation in the tourism sector.*

## 3. Data and Variables

This study used a sample of 120 tourism companies from 26 countries across five continents. The data spanned the period of 2002 to 2019. This period was chosen because these years corresponded to the earliest and most recent years for which environmental, social and governance (ESG) data were available at the time of data collection. These tourism companies are publicly listed, and on 25 March 2020, they had at least 4 years of available data. The selection criterion of publicly listed status was applied because data availability is better for listed companies. Moreover, the implementation of environmental initiatives requires substantial investment in technology, infrastructure and human resources, and listed companies have better access to funds for such practices than unlisted companies [111]. A minimum time span of 4 years was chosen because four periods are required to provide panel data that enable the use of lags of the explanatory variables as instruments. This feature is essential for the econometric model to verify the existence of endogeneity and to correct for this issue. In addition, this criterion also provided a more balanced panel.

The selected companies were drawn from an initial sample of 399 listed companies in the Thomson Reuters Eikon database [112]. Of these, 279 were discarded because they did not have at least 4 years of data on environmental performance. This data source was selected because it provides rich data covering financial as well as ESG data.

According to the sector classification proposed by Refinitiv [113], the sampled companies can be divided into four subsectors: restaurants and bars, leisure and recreation, casinos and gaming, and hotels, motels and cruises. Table 2 presents the distribution of the sampled companies by continent and country.

### 3.1. Dependent Variables

To measure the environmental performance of tourism companies, four indicators from the Thomson Reuters Eikon [112] database were used. This database provides scores on the three dimensions of ESG (aggregated indicators) and their underlying categories, as well as information on the items used to calculate these scores. Thanks to these rich data, studies such as those by Kyaw et al. [25] and Birindelli et al. [32] have used the score of the environmental and social dimensions as the dependent variable. The scores for the pillars of the aggregate measures have been used to a lesser extent in the CSR literature [47,51,85]. However, these pillars are important and should be considered, given that using only the aggregate indicators for each dimension could mean that relevant information is overlooked.

Therefore, in this study the dependent variables were taken as the overall environmental performance score and its three categories: resource use (20 items), emissions (28 items) and innovation (20 items). This approach lent robustness to the results, thanks to the use of various measures, while helping identify the importance of each of the areas of environmental performance. This study thus addresses the shortcomings mentioned earlier. To help identify the information covered by the environmental measures, Table 3 shows the labels assigned to these four variables, their definition and their content.

Finally, regarding the method, calculation of the environmental score involved obtaining the relative value for each category and then calculating the overall index. Therefore, the starting point was to determine the score for each category as a weighted average of the groups of data for the industry that comprise it. Accordingly, the weighting for each group varied by industry. These data were previously generated by assigning a score to each of the areas measured by the category. Proxies were used where it was not possible to find data for a specific variable. The category scores were normalised and expressed as percentages ranging from 0 to 100. Finally, the score for the environmental pillar was calculated using the sum of the values resulting from the weightings of its three categories. The resource use and emissions categories were given a weighting of 35%, and the innovation category was given a weighting of 29% [114].

Thus, the four environmental variables used in this study took values ranging from 0 to 100, where 0 indicates the worst possible environmental performance and 100 denotes the best possible environmental performance [87].

### 3.2. Independent Variables

This section presents and justifies the choice of independent variables. These variables can be divided into two groups. The first consists of variables of interest used to address the research aims. The second group consists of control variables included to ensure that the econometric model was correctly specified. The data used to create these variables were gathered from the Thomson Reuters Eikon database [112]. On the whole, the variables of interest for this study were the board gender diversity variables that have most commonly been used in studies of corporate environmental performance, as detailed in Table 4.

A large number of gender variables were selected. In fact, this study used more such variables than many other studies of the influence of board gender diversity on environmental performance [50,82,83]. This large number of variables lends robustness to the empirical evidence.

The rationale for using these gender measures is that several studies have reported that women are more likely to engage in socially responsible practices given their greater focus on stakeholders [73], and in particular, on environmental sustainability [18]. However, scholars disagree over the minimum number of women board members required in order for them to exert a noticeable influence on corporate performance [67,68], as described by critical mass theory [59]. These reasons, together with the limited evidence of the impact of a critical mass of women directors on environmental sustainability [32], motivated the decision to include the dichotomous variables *Dum1*, *Dum2* and *Dum3*.

The variable *Dum1* identifies whether merely having at least one woman director plays a relevant role in environmental performance. For example, Shoham et al. [18] showed that companies whose boards of directors had at least one woman were more predisposed towards environmental sustainability. Similarly, He and Jiang [72] and Atif et al. [89] have reported that having at least two women on the board of directors is sufficient for there to be a noticeable female influence on environmental performance and renewable energy consumption, respectively, hence the reason for including *Dum2*. However, a higher proportion of studies have shown that companies with at least three female directors can ensure that women have a stronger role on the board [57,115], pay more attention to environmental issues [64,70] and face less environmental litigation [115]. Hence, *Dum3* was included in the model. In addition, some authors, such as Kyaw et al. [25] and Shoham et al. [18], do not subscribe to critical mass theory. Instead, they suggest that a minimum level of female representation is not necessary for women to promote socially and environmentally responsible actions.

The variables *Dum30* and *Dum40* were included to control for the possibility of significant differences in business performance between companies with at least 30% and 40% of women directors, respectively, and companies that did not meet these thresholds. The aim was to explore the possible repercussions of corporate governance gender quotas, such as those proposed by the European Parliament and Council Directive [119]. Regarding the arguments in the literature, Ahern and Dittmar [120] reported that restructuring the board of directors by including women on the board to comply with gender quotas lowers the value of the company. However, according to Birindelli et al. [32], achieving 30% female representation on the board reflects a change in the trend of environmental performance, with the sign depending on the gender of the chief executive officer (CEO). This change is negative when the CEO is male, but positive when the CEO is female. Gallego-Sosa et al. [95] reported that achieving a minimum of 40% women directors does not affect environmental performance. In contrast, Lafuente and Vaillant [121] reported that a heterogeneous board in terms of gender, composed of 40% to 60% women (a balanced group according to Kanter), leads to better company performance. Crucially, these measures are novel in gender and corporate governance studies [117]. They have only been used in environmental research by Gallego-Sosa et al. [95].

The variables *Nwom* and *Pwom* are also essential. The variable *Nwom* was included because socially responsible companies have more women than non-socially responsible companies [31,62]. In addition, the number of women on the board of directors has been found to have a positive influence on environmental performance by increasing the implementation of environmental practices [64,84] such as those related to the use of renewable energy [89]. The level of female representation on the board of directors (*Pwom*) is also important because a higher proportion of women directors positively affects corporate environmental performance [24,25,87]. This influence is explained by the implementation of environmental strategies [86] and environmental practices [47]. Such environmental strategies include those related to the reduction of resource use and emissions [27,85], the formation of strategic alliances in renewable energy [82] and environmental innovation measured in terms of patents [91].

Finally, the variable *Blau* was included. This index is the most widely used diversity index in the literature, with several studies citing it as a good measure of diversity [26,122]. Lu and Herremans [26] and Burkhardt et al. [47] reported that board gender diversity positively influences environmental performance. The values of the Blau index range from 0 to 0.5, where 0 indicates the lowest possible diversity due to the absence of women directors, and 0.5 indicates the maximum diversity when the number of female board members equals the number of male board members. This index was calculated using the following formula [118]:

$$\;Blau\;index=1-\overset{n}{\underset{i=1}{\sum }}P_{i}^{2}$$

where $P_{i}$ is the percentage of men or women on the board of directors and n is the number of categories (i.e., male and female).

The second group of independent variables consists of the control variables, which are presented in Table 5. These variables were selected because their influence on environmental performance receives support in the literature, as indicated by the cited studies. Six control variables were used. Four were measures of corporate governance (*IndepMem*, *AveTen*, *EnvTra* and *Ndir*), and two were financial indicators, namely the size of the company (*Temp*) and financial performance (return on assets or *ROA*).

First, the degree of independence of the board was included in the model, measured as the percentage of independent directors (*IndepMem*). Several studies have shown the positive influence of board independence on the number of green initiatives [72] and environmental performance [47,111]. One explanation for this finding is that in addition to reducing agency costs, independent members have a greater focus on long-term actions, stakeholder needs [125] and environmental compliance [126].

The *AveTen* and *EnvTra* variables were included because of the scarcity of research on the influence of board members’ experience on business performance [123] and the importance of environmental management training in corporate environmental action [95], respectively. Research is lacking in this area, even though board members’ experience and education are closely related to business performance [127] and hence environmental performance [101].

The variable *Ndir* (i.e., the number of board members) is also important. Although some studies suggest that smaller boards are more effective, participatory and cohesive [128], larger boards are widely accepted to have greater diversity and access to information [129]. For instance, several studies have shown that board size positively influences the implementation of CSR best practices [49], particularly environmental performance [25,129].

Finally, larger companies have better environmental performance [87] because they are more environmentally aware [83]. Therefore, the variable *Temp* was included. This measure is often used in the business performance literature as indicative of firm size [72,116]. Similarly, return on assets (*ROA*) is a critical factor in environmental performance because it influences whether companies will have the resources and capabilities needed for more effective environmental initiatives [130]. Accordingly, several authors, such as Kyaw et al. [25] and Naciti [44], have reported that companies with higher ROA have better environmental performance.

## 4. Method

To achieve the research aims, as in previous studies [25,27,87,117], econometric estimation of static linear equations was performed. These equations are defined below:

$${\mathrm{EnvSc}}_{\mathrm{it}}=\beta_{1}+\beta_{2}\;{\mathrm{Gen}}_{\mathrm{it}}+\beta_{3}\;{\mathrm{IndepMem}}_{\mathrm{it}}+\beta_{4}\;{\mathrm{AveTen}}_{\mathrm{it}}+\beta_{5}\;{\mathrm{EnvTra}}_{\mathrm{it}}+\beta_{6}\;{\mathrm{Ndir}}_{\mathrm{it}}+\beta_{7}\;{\mathrm{Temp}}_{\mathrm{it}}+\beta_{8}\;{\mathrm{ROA}}_{\mathrm{it}}+\;\tau_{t}+{ɛ}_{\mathrm{it}}$$

$${\mathrm{ResUseSc}}_{\mathrm{it}}=\beta_{1}+\beta_{2}\;{\mathrm{Gen}}_{\mathrm{it}}+\beta_{3}\;{\mathrm{IndepMem}}_{\mathrm{it}}+\beta_{4}\;{\mathrm{AveTen}}_{\mathrm{it}}+\beta_{5}\;{\mathrm{EnvTra}}_{\mathrm{it}}+\beta_{6}\;{\mathrm{Ndir}}_{\mathrm{it}}+\beta_{7}\;{\mathrm{Temp}}_{\mathrm{it}}+\beta_{8}\;{\mathrm{ROA}}_{\mathrm{it}}+\;\tau_{t}+{ɛ}_{\mathrm{it}}$$

$${\mathrm{EmiSc}}_{\mathrm{it}}=\beta_{1}+\beta_{2}\;{\mathrm{Gen}}_{\mathrm{it}}+\beta_{3}\;{\mathrm{IndepMem}}_{\mathrm{it}}+\beta_{4}\;{\mathrm{AveTen}}_{\mathrm{it}}+\beta_{5}\;{\mathrm{EnvTra}}_{\mathrm{it}}+\beta_{6}\;{\mathrm{Ndir}}_{\mathrm{it}}+\beta_{7}\;{\mathrm{Temp}}_{\mathrm{it}}+\beta_{8}\;{\mathrm{ROA}}_{\mathrm{it}}+\;\tau_{t}+{ɛ}_{\mathrm{it}}$$

$${\mathrm{InnoSc}}_{\mathrm{it}}=\beta_{1}+\beta_{2}\;{\mathrm{Gen}}_{\mathrm{it}}+\beta_{3}\;{\mathrm{IndepMem}}_{\mathrm{it}}+\beta_{4}\;{\mathrm{AveTen}}_{\mathrm{it}}+\beta_{5}\;{\mathrm{EnvTra}}_{\mathrm{it}}+\beta_{6}\;{\mathrm{Ndir}}_{\mathrm{it}}+\beta_{7}\;{\mathrm{Temp}}_{\mathrm{it}}+\beta_{8}\;{\mathrm{ROA}}_{\mathrm{it}}+\;\tau_{t}+{ɛ}_{\mathrm{it}}$$

*EnvSc* is the overall environmental score. *ResUseSc* is the resource use score. *EmiSc* is the emissions score. *InnoSc* is the innovation score. *Gen* corresponds to each of the eight selected gender measures. *IndepMem* refers to the percentage of independent board members. *Ant* is the average number of years for which board members remain on the board. *EnvTra* is a dummy variable that takes the value 1 if employees receive green training, and 0 otherwise. *Ndir* is the number of board members. *Temp* is the number of employees at the company. *ROA* is the return on assets. The term $\varepsilon_{it}$ is the random error term for firm i in period t. Finally, $\tau_{t}$ is a cumulative dummy variable representing time fixed effects. This variable was included to control for unobservable factors that may influence the behaviour of the dependent variable over time.

As these equations are static linear equations based on panel data, they can be estimated using two procedures: a fixed effects model or a random effects model. The choice of model is based on verification of the assumption of the absence of correlation between the unobservable individual effect and the explanatory variables. To do so, the Hausman test is used. If the results of this test lead to the rejection of the hypothesis of absence of correlation, the only consistent estimator is the fixed effects estimator. Conversely, if this hypothesis is not rejected, both estimators are consistent, but the random effects estimator is the efficient estimator [131]. The results of the Hausman test, displayed under “*p* value (Hausman: FE/RE)” in Tables 7–10, reveal the existence of correlation between the explanatory variables and the individual unobservable effect. Therefore, the fixed effects model was used.

The next step required verification of the assumption of exogeneity of the independent variables. This condition is essential for the fixed effects estimator to be consistent [131]. The evidence indicates possible endogeneity problems in the model. This possible endogeneity can be explained by a relationship of bidirectional causality between the dependent variable and the explanatory variables [83,115] or by the omission of relevant variables [35]. Therefore, the Hausman test was used to verify that the hypothesis of zero correlation between the explanatory variables and the error term was met in all cases (exogeneity assumption). The data in Tables 7–10 under “*p* value (Hausman: FEIV/FE)” reflect this result. For this test, the first lags of the explanatory variables were used as instruments [132].

Finally, estimation was performed using a variance–covariance matrix of errors that were robust to heteroscedasticity between individuals and to serial correlation of the errors of a given individual.

## 5. Results and Discussion

### 5.1. Description of the Sample

Before presenting the results of the analysis, this section offers a characterisation of the sample by presenting descriptive statistics for the variables used in the study (Table 6). The measures of interest in this study were the environmental performance and gender diversity indicators.

The statistics relating to the environmental variables reveal low scores overall and by category. The arithmetic mean is below the midpoint score (50) for each of the four measures. For the three categories within the overall index, the highest mean (37.13) corresponds to the reduction of resource use (*ResUseSc*). This value is higher than the corresponding value for the overall score (*EnvSc*). The mean score for the reduction of emissions category (*EmiSc*) is only a few points lower (34.61). The lowest mean value (8.96) is for innovation (*InnoSc*), which is considerably lower than the rest. The four environmental scores have a strikingly wide range, as reflected by the minimum and maximum values. This wide range is consistent with the high dispersion of the observations with respect to the aforementioned means. This dispersion is greater than 30 points for *EnvSc*, *ResUseSc* and *EmiSc*, and is equal to 22.11 points for *InnoSc*.

In line with these data, the percentiles show the poor environmental commitment of the sampled companies and the significant difference between the innovation category and the categories of reduction of resource use and emissions. For example, although 50% of the observations (P50) in the *ResUseSc* and *EmiSc* categories are below 33.56 and 26.75, respectively, 75% of the observations for *InnoSc* are equal to zero (P75).

Analysis of the gender variables shows that some companies did not have women directors in the period analysed (see Min for *Nwom*). Female representation is low and reflects little gender diversity in the sampled firms. Specifically, the average percentage of female directors is only 15.3% (see *Pwom*), with 75% of the observations for this variable taking a value of less than 22.2% (see P75). Although there is at least one case in which women have a representation of 57.1% (see Max for *Pwom*) and therefore outnumber the male directors, the data show that gender parity at the top management level is still a distant goal. For example, female representation on the board is at least 30% for only 11.9% of observations (*Dum30*), and only 3.6% of observations have at least 40% female representation (*Dum40*). Similar results, which imply a clear under-representation of women on the boards of tourism companies, have already been reported by Barrientos-Báez et al. [133] and Uyar et al. [24]. One possible reason for this finding is that women’s career development in the tourism sector is limited, with women finding it difficult to access management positions [16].

With respect to the remaining measures, starting with the governance variables, nearly 60% of board members are independent (*IndepMem*). On average the directors have been on the board for almost 8 years (*AveTen*). Finally, 40.2% of the observations indicate that the sampled tourism companies have environmental training policies (*EnvTra*). The number of employees at these companies ranges massively, from 15 to 508,714 (*Temp*). The sampled tourism companies are profitable, with an average return on assets (*ROA*) of 7.76%. In addition, more than 75% of the observations indicate positive profitability (P25).

In conclusion, despite policies and practices for the reduction of resource use and emissions and for environmental innovation through the development of processes and products designed to help care for the planet, considerable efforts can still be made in all three categories, especially innovation. This situation may stem from a lack of knowledge and interest in environmental protection among directors, as suggested by Erdogan and Baris [134] in their study of a sample of hotels, given that most of the tourism companies analysed in this study have not adopted environmental management training policies (see *EnvTra*, Table 6).

Regarding gender diversity, the under-representation of women on the boards of global tourism companies is notable. The evidence suggests that the tourism sector is some way from attaining the minimum threshold of 40% representation of women on the board recommended by the European Commission in its directive to improve the gender balance among the non-executive directors of listed companies [119].

### 5.2. Econometric Analysis: Board Gender Diversity and Environmental Performance

This section presents the results of the econometric estimation of the equations stated earlier (Table 7, Table 8, Table 9 and Table 10). The aim is to provide statistical evidence of the influence of gender diversity on environmental performance, both generally and specifically in terms of less resource use, lower emissions and environmental innovation. This analysis thus addresses the research aims of the present study. An equation was estimated for each dependent variable and each of the eight proposed gender measures. The models were statistically significant at the 99% confidence level in all cases, as reflected by the *p* value of the F-test, “*p* value (F)”.

The relationship of interest in this analysis was the link between gender diversity and environmental performance. Focusing on this relationship, the results indicate a number of interesting findings. First, for the estimates where the dependent variables were *EnvSc*, *ResUseSc* and *EmiSc* (Table 7, Table 8 and Table 9), four of the eight gender measures (*Dum3*, *Nwom*, *Pwom*, *Blau*) had statistically significant coefficients at a confidence level of 95 or 99%. In all cases, these coefficients were negative. Only in the estimation for *ResUseSc* was the coefficient for *Dum30* statistically significant at the 90% confidence level. This coefficient was also negative. Second, in the case of the dependent variable *InnoSc*, the results clearly differ from those for the other dependent variables. Only the coefficients associated with the variables *Dum30* and *Nwom* were statistically significant at the 90% and 95% confidence level, respectively (Table 10). Both of these coefficients were positive. Finally, none of the coefficients associated with the variables *Dum1*, *Dum2* or *Dum40* were statistically significant (Table 7, Table 8, Table 9 and Table 10).

Therefore, these results provide statistical evidence of a negative influence of board gender diversity on corporate environmental performance. This result is robust because it emerged in several estimates with different dependent variables. Specifically, an increase in the number of women on the board to at least three (*Dum3*), an increase in the number of women on the board (*Nwom*), an increase in the proportion of women (*Pwom*) and greater gender equality among directors (*Blau*) result in a lower environmental performance score (both overall and in terms of reduced resource use and emissions).

According to the estimates for the dependent variable *InnoSc*, the statistical significance of the positive coefficients for *Dum30* and *Nwom* provides evidence that the score for process and product innovation to help care for the planet is greater in companies with at least 30% female representation on the board and increases as the number of women on the board increases.

Third, the statistical evidence from all estimates also indicates no differences in terms of environmental performance between companies without female directors and companies with women on the board (see *Dum1* coefficients), between companies with at least two women on the board and those with fewer than two (see *Dum2* coefficients), and between companies with a minimum representation of 40% women on the board and those that do not meet this threshold (see *Dum40* coefficients).

Consequently, the search for corporate governance mechanisms that influence corporate environmental performance reveals a generally negative relationship in terms of gender diversity on the board of directors. This result is supported by previous studies, which report a decrease in the adoption of environmental practices and environmental policies as the presence of women directors increases [30,31,93,94].

The literature offers several explanations for this negative influence. The first reason is the weak, limited role of women due to their under-representation on the board [135], which is the case in the present sample (Table 6). Second, the presence of women on the board means greater board diversity. This greater diversity may lower firm performance in terms of caring for the planet because of increased conflict and reduced cooperation among board members due to the heterogeneity of the board [40,136]. Finally, the presence of women on the board may be the result of a token gesture solely to improve the corporate image. This situation would limit the scope for women directors to act, preventing their actions from enhancing the firm’s environmental performance [30].

However, many studies have shown that companies with greater board gender diversity adopt more actions and strategies aimed at environmental sustainability [24,43,50,73,137]. The argument is that women board members focus more on social and environmental issues [47,138,139]. Therefore, the evidence provided by this study contradicts studies that indicate that board gender diversity is positively related to environmental performance [44,86,87,88] and reduced resource use and emissions [27,85,140]. These discrepancies can be explained by the complex relationship between board gender diversity and responsible practices [141], which may be determined by the unique characteristics of the context [142]. These characteristics include the company’s human resources policy [143], the existence of a CSR committee [144], CEO gender [32], ownership [91] and sector [79,145].

Environmental innovation is the only performance measure with a positive relationship with gender diversity (albeit for only two of the eight estimates). Several arguments support these results. First, there is a stronger relationship between the presence of women and environmental innovation than with the other environmental measures considered in this study. Accordingly, the low female representation does not interfere with the positive effect of women’s presence on environmental innovation initiatives [95]. Moreover, the presence of women on the board has certain benefits, such as greater innovation and creativity [60,146]. If these benefits outweigh the internal conflicts and other such negative effects arising from this greater board diversity [147], then environmental innovation may actually be enhanced. This result is supported by several studies, which have shown that companies with greater female board representation are more likely to engage in environmental innovation practices [90] and to implement a greater number of such practices [72,92], especially when the female directors are also independent [91].

This study also supports critical mass theory, with the results confirming the need for there to be at least three women on the board for these female directors to influence environmental actions. In a study of environmental litigation, Liu [115] reported similar findings, although the influence in the present study is negative. This finding can be explained by the fact that the presence of at least one or two women on the board is not enough to ensure that they can influence environmental performance [145]. However, there are discrepancies regarding this theory. For instance, some studies suggest that two women constitute a critical mass. According to these studies, the presence of at least two women on the board is sufficient in order for them to exert a significant influence in several areas, such as the disclosure of information on GHG emissions [58] and environmental innovation [72]. However, Kyaw et al. [25] argued that a critical mass of women on the board is not necessary for better environmental performance, suggesting instead that the presence of one woman on the board is the only relevant factor.

Furthermore, the empirical evidence fails to confirm the need for at least 40% female representation on the board of directors. This percentage has been proposed by the European Parliament and Council Directive [119]. Nevertheless, Fernández-Torres et al. [117] showed that attaining this minimum proportion of women directors is a differentiating factor in corporate performance in the financial sector. However, the results may be due to the low proportion of cases (only 3.6%) where this condition is met (see Table 6). Thus, the desired effect could not be observed.

According to the results, environmental performance is also conditioned by variables other than gender. Specifically, overall environmental performance, resource use and emissions reductions, and environmental innovation are positively influenced by policies to provide environmental management training to employees. Companies with larger boards have better overall environmental performance, as well as adopting a greater number of policies on resource use and emissions reductions. Furthermore, these three environmental performance variables are also positively related to the average tenure of board members. However, tourism companies perform worse in environmental innovation as the percentage of independent board members increases. Finally, higher ROA leads to greater innovation.

To conclude, for the most part, hypotheses *H1*, *H2* and *H3* are not supported. The results of the analysis reveal a different relationship from the predicted relationship in the estimates for three of the four dependent variables. However, the significant positive coefficients in the estimates using environmental innovation as the dependent variable provide support for hypothesis *H4*.

## 6. Conclusions

This study examined 120 listed companies from the global tourism sector over the period 2002 to 2019. The aim was to analyse whether board gender diversity conditions these companies’ environmental performance. The analysis, which was based on a fixed effects model, leads to the following conclusions.

The empirical evidence essentially shows that a greater female presence on the board is associated with worse environmental performance. This finding holds for the case of both overall environmental performance and the specific categories of policies and practices related to reducing the use of resources and lowering emissions. The analysis provides robust evidence of this negative influence, which may result from the internal conflicts that arise due to the greater range of opinions and perspectives in gender-diverse groups. Consequently, it is more difficult to reach a consensus on critical decisions. This lack of consensus leads to less effective decision making. Moreover, judging by the low representation of women, any discrepancies that arose in the companies studied would probably be resolved in favour of men (i.e., the dominant group), thus under-utilising female talent.

However, the opposite relationship was observed for environmental innovation in two of the eight gender measures. According to this relationship, the performance of tourism firms improves as the number of female board members increases. This finding can be explained by the positive differential effect of female board members on innovation, which may overlap with the above-mentioned conflicts in the decision-making process. This study also supports critical mass theory by showing the need to achieve female representation of at least three women in order for female directors to exert a significant influence on corporate environmental performance.

This research is valuable given the lack of studies that address gender issues in management and their effect on environmental performance in the tourism sector. The findings of this study have practical implications for managers of tourism companies, political decision makers and legislators. First, the under-representation of women on the boards of companies in the tourism sector indicates the existence of a glass ceiling for women in the corporate hierarchy. This limitation has consequences for companies in terms of their environmental performance. This situation highlights the need for regulations in tourism and the ongoing support of diversity education in society to empower women and secure equal conditions for women in the workplace. Specifically, the recommendation is for the implementation of gender quotas at the board level, sanctions for pay gaps between men women, and work-life balance policies to help reconcile work commitments with maternity, childcare and family obligations. Similarly, incentives through tax policies or social recognition should be provided to firms that offer promotions to women. At the same time, tourism managers’ commitment to gender diversity is essential. Firms should implement human resource policies based on equal opportunities for men and women that cover all processes, from selection and training to promotion and compensation.

In addition, the lack of involvement of these organisations in caring for the planet is a warning sign for entrepreneurs in this sector, who must increase their awareness and implementation of environmental practices given that their activity depends heavily on the natural resources of the local area. Accordingly, the managers of these firms should create programmes to raise awareness and manage their environmental impact. Such programmes represent the only way to create policies and actions that contribute to corporate environmental education and that build collective awareness and involvement in terms of the need to care for the environment. This care for the environment offers the only way to guarantee access to the resources that this sector depends on for generations to come.

Therefore, there is a need for greater involvement from public administrations, which have a duty to encourage the implementation of these practices through environmental policies and incentives. Such policies should entail sanctions for the abuse of natural resources, the control and reduction of pollution, and the enforcement of reporting on environmental impact and the measures taken to curb this impact. Likewise, improvements in firms’ environmental behaviour can be promoted through tax incentives for meeting certain environmental goals or through recognition for best practices in this area, thereby enhancing the corporate image of these firms.

Finally, this study has some limitations. The first limitation relates to the lack of data availability for most of the tourism firms identified in the initial search. This lack of data availability meant that many of these firms were omitted from the study. Another difficulty stems from the use of composite indicators for the environmental performance scores. The use of such indicators can mask important information. Furthermore, the analysis did not control for country effects. Doing so may be important given the cultural diversity and the range of gender and environmental regulations across different countries. Finally, this study did not consider qualitative aspects such as the skills and values of female directors because of the complexity involved in measuring these factors. Analysis of these aspects may help shed light on the relationship between board gender diversity and environmental performance.

One proposal is for future research to use other environmental performance variables that measure more specific elements. Doing so could improve the specification of certain practices. For example, it would be of interest to focus on specific topics addressed in the SDGs relating to environmental issues. The aim would be to obtain indicators that exclusively reflect information on certain items relating to one or more of the targets within a given SDG. Doing so could reveal the involvement of companies in achieving these crucial global goals, as well as providing evidence of the influence of gender diversity in achieving these SDGs. Similarly, it would be of interest to perform analysis with a geographical focus to control for the characteristics of each individual country or region. Finally, other qualitative variables such as the age, education and experience of women managers should be studied as gender variables.

## Figures and Tables

**Table 1:** Review of studies of the relationship between gender diversity and environmental performance.

Authors	Performance Variable	Results
[18,24,25,26,27,32,42,43,44,47,50,51,64,70,73,79,82,83,84,85,86,87,88]	Environmental performance	Positive relationship
[27,47,85,89]	Use of resources	
[27,71,85]	Emissions	
[19,47,72,85,90,91,92]	Environmental innovation	
[30,31,93,94]	Environmental performance	Negative relationship
[87]	Consumption of water and energy	
[95]	Environmental innovation	
[28,34,96,97,98,99,100,101,102,103,104,105,106]	Environmental performance	No relationship
[29]	Consumption of energy	
[27,85]	Environmental innovation	

Source: authors, based on the literature review.

**Table 2:** Distribution of the sampled companies by continent and country.

Continent	Country	Number of Companies
Africa		3
	South Africa	3
America		53
	Canada	4
	United States	49
Asia		25
	China	1
	Republic of Korea	2
	Philippines	1
	Hong Kong	7
	Japan	4
	Macao	3
	Malaysia	3
	Singapore	1
	Sri Lanka	1
	Thailand	1
	Taiwan	1
Europe		25
	Germany	1
	Spain	1
	France	3
	Gibraltar	1
	Greece	1
	Ireland	1
	Isle of Man	1
	Italy	1
	Malta	1
	United Kingdom	14
Oceania		14
	Australia	12
	New Zealand	2
Total		120

Source: authors, based on Refinitiv [112].

**Table 3:** Environmental performance variables.

Label	Definition	Content
*EnvSc*	General environmental performance score	Uses information provided by each company on its ability to operate with an efficient use of natural resources, the reduction of emissions, and innovation and support for the development of eco-efficient products and services
*ResUseSc*	Resource use score	Captures the performance and ability of each company to reduce the use of materials, energy and water and to find more eco-efficient solutions by improving supply chain management
*EmiSc*	Emissions reduction score	Measures the commitment and effectiveness of each company in reducing emissions in production and operational processes
*InnoSc*	Innovation score	Captures the ability of each firm to reduce its environmental impact and create new market opportunities through new environmental technologies or processes or ecologically designed products

Source: authors, based on Refinitiv [112].

**Table 4:** Gender diversity variables.

Label	Definition	Supporting Literature
*Dum1*	Dummy taking a value of 1 if there is at least one woman on the board of directors, and 0 otherwise	[18,25,72,80,85,89]
*Dum2*	Dummy taking a value of 1 if there are at least two women on the board of directors, and 0 otherwise	[58,72,89]
*Dum3*	Dummy taking a value of 1 if there are at least three women on the board of directors, and 0 otherwise	[18,32,70,89,115,116]
*Dum30*	Dummy taking a value of 1 if there are at least 30% of women on the board of directors, and 0 otherwise	[95]
*Dum40*	Dummy taking a value of 1 if there are at least 40% of women on the board of directors, and 0 otherwise	[95,117]
*Nwom*	Number of female board members	[29,31,64,72,84,89]
*Pwom*	Proportion of women directors, calculated as the ratio of the number of women on the board to the total number of board members	[24,27,47,50,51,73,85,87,97]
*Blau*	Index of gender diversity on the board of directors [118]	[26,47,80,103]

Source: authors, based on a review of the studies cited in the table.

**Table 5:** Control variables.

Label	Definition	Supporting Literature
*IndepMem*	Percentage of independent board members	[25,47,72,105]
*AveTen*	Average number of years that board members have held the position	[30,123,124]
*EnvTra*	Dummy taking a value of 1 if environmental management training policies are implemented, and 0 otherwise	[95]
*Ndir*	Number of board members	[26,102,103,116]
*Temp*	Number of full-time employees at the end of the tax period	[31,44,64,72,116]
*ROA*	Return on assets: ratio of profit after tax to average assets for the tax year	[26,64,102,111]

Source: authors, based on a review of the studies cited in the table.

**Table 6:** Descriptive statistics of the variables (2002–2019).

Variables	Arithmetic Mean	SD	Min	Max	P25	P50	P75	N
*EnvSc*	33.878	30.327	0	96.255	2.122	28.193	62.640	1198
*ResUseSc*	37.137	33.835	0	99.615	0	33.566	67.171	1198
*EmiSc*	34.617	34.110	0	99.653	0	26.752	66.463	1198
*InnoSc*	8.967	22.110	0	97.222	0	0	0	1198
*Dum1*	0.792	0.406	0	1	1	1	1	1170
*Dum2*	0.425	0.494	0	1	0	0	1	1170
*Dum3*	0.289	0.453	0	1	0	0	1	1170
*Dum30*	0.119	0.325	0	1	0	0	0	1170
*Dum40*	0.036	0.188	0	1	0	0	0	1170
*Nwom*	1.504	1.268	0	7	1	1	2	1170
*Pwom*	0.153	0.118	0	0.571	0.083	0.143	0.222	1170
*Blau*	0.231	0.151	0	0.5	0.153	0.245	0.345	1170
*IndepMem*	59.298	22.367	0	100	44.444	62.202	76.923	1174
*AveTen*	7.61	4.049	0	24.75	4.662	6.853	9.952	1160
*EnvTra*	0.402	0.49	0	1	0	0	1	1199
*Ndir*	9.672	2.96	2	26	8	9	11	1194
*Temp*	37479.5	76565.7	15	508714	3400	11219.5	31000	1318
*ROA*	7.7607	12	−64.31	204.98	2.719	6.124	10.905	1830

Source: authors, based on Refinitiv [112]. Compiled using Stata 16, StataCorp LLC, Badajoz, Spain. SD = standard deviation; Min = minimum value; Max = maximum value; P25 = 25th percentile; P50 = 50th percentile; P75 = 75th percentile; N = number of observations.

**Table 7:** Dependent variable: *EnvSc*. Fixed effects estimator (2002–2019).

Variables	(1)	(2)	(3)	(4)	(5)	(6)	(7)	(8)
*Dum1*	−1.7955							
*Dum2*		−2.7040						
*Dum3*			−4.9474 ***					
*Dum30*				−3.0859				
*Dum40*					−4.2413			
*Nwom*						−2.9679 ***		
*Pwom*							−31.7088 ***	
*Blau*								−20.2395 **
*IndepMem*	−0.0328	−0.0255	−0.0204	−0.0199	−0.0244	−0.0294	−0.0225	−0.0277
*AveTen*	2.2317 ***	2.2090 ***	2.1423 ***	2.2755 ***	2.2645 ***	2.0953 ***	2.1098 ***	2.1408 ***
*EnvTra*	15.2397 ***	15.1454 ***	15.097 ***	14.8587 ***	14.9088 ***	14.9823 ***	14.4767 ***	14.6851 ***
*Ndir*	1.4355 **	1.4437 **	1.3718 **	1.5732 **	1.5383 **	1.8494 ***	1.5794 **	1.5765 **
*Temp*	−0.9220	−0.7906	−0.7563	−1.2384	−1.1067	−1.0681	−1.1805	−1.0981
*ROA*	−0.0759	−0.0764	−0.0713	−0.0770	−0.0808	−0.0052	−0.0543	−0.0573
N	851	851	851	851	851	851	851	851
R^2^ (within)	0.6024	0.6035	0.6081	0.6035	0.6030	0.6114	0.6116	0.6090
*p* value (F)	0.0000	0.0000	0.0000	0.0000	0.0000	0.0000	0.0000	0.0000
*p* value (Hausman: FE/RE)	0.0000	0.0000	0.0000	0.0000	0.0000	0.0000	0.0000	0.0000
*p* value (Hausman: FEIV/FE)	0.9977	0.9966	0.9987	0.9962	0.9997	0.8784	0.7285	0.8484

Source: authors, based on Refinitiv [112]. Compiled using Stata 16, StataCorp LLC, Badajoz, Spain. *** significant at the 99% level, ** significant at the 95% level. R2 (within): coefficient of determination of the transformed model. *p* value (F): *p* value of the test of model significance. *p* value (Hausman: FE/RE): *p* value of the Hausman test under the null hypothesis of absence of correlation between the explanatory variables and the individual unobservable effect. *p* value (Hausman: FEIV/FE): *p* value of the Hausman test under the null hypothesis of absence of correlation between the explanatory variables and the error term. Time dummies are omitted for reasons of brevity and practicality. The estimation was performed with errors that are robust to heteroscedasticity and autocorrelation.

**Table 8:** Dependent variable: *ResUseSc*. Fixed effects estimator (2002–2019).

Variables	(1)	(2)	(3)	(4)	(5)	(6)	(7)	(8)
*Dum1*	−1.9426							
*Dum2*		−2.7762						
*Dum3*			−5.0060 **					
*Dum30*				−4.4023 *				
*Dum40*					−3.9992			
*Nwom*						−3.0442 ***		
*Pwom*							−33.1578 ***	
*Blau*								−20.7839 **
*IndepMem*	−0.0639	−0.0561	−0.0511	−0.0485	−0.0554	−0.0602	−0.0534	−0.0587
*AveTen*	2.0655 ***	2.0436 ***	1.9768 ***	2.0968 ***	2.0905 ***	1.9271 ***	1.9267 ***	1.9618 ***
*EnvTra*	15.9634 ***	15.8663 ***	15.8188 ***	15.6184 ***	15.7519 ***	15.3052 ***	15.2869 ***	15.5114 ***
*Ndir*	1.3019 **	1.3069 **	1.2331 **	1.4259 **	1.3684 **	1.7230 **	1.4130 **	1.4091 **
*Temp*	−1.5478	−1.4137	−1.3811	−1.9061	−1.7038	−1.6985	−1.7839	−1.6974
*ROA*	−0.0755	−0.0764	−0.0712	−0.0752	−0.0811	−0.0511	−0.0533	−0.0568
N	851	851	851	851	851	851	851	851
R^2^ (within)	0.5285	0.5295	0.5336	0.5308	0.5289	0.5370	0.5374	0.5347
*p* value (F)	0.0000	0.0000	0.0000	0.0000	0.0000	0.0000	0.0000	0.0000
*p* value (Hausman: FE/RE)	0.0000	0.0000	0.0000	0.0000	0.0000	0.0000	0.0000	0.0000
*p* value (Hausman: FEIV/FE)	0.9942	0.9987	0.9995	0.9995	0.9957	0.9047	0.7756	0.7734

Source: authors, based on Refinitiv [112]. Compiled using Stata 16, StataCorp LLC, Badajoz, Spain. *** significant at the 99% level, ** significant at the 95% level, * significant at the 90% level. R2 (within): coefficient of determination of the transformed model. *p* value (F): *p* value of the test of model significance. *p* value (Hausman: FE/RE): *p* value of the Hausman test under the null hypothesis of absence of correlation between the explanatory variables and the individual unobservable effect. *p* value (Hausman: FEIV/FE): *p* value of the Hausman test under the null hypothesis of absence of correlation between the explanatory variables and the error term. Time dummies are omitted for reasons of brevity and practicality. The estimation was performed with errors that are robust to heteroscedasticity and autocorrelation.

**Table 9:** Dependent variable: *EmiSc*. Fixed effects estimator (2002–2019).

Variables	(1)	(2)	(3)	(4)	(5)	(6)	(7)	(8)
*Dum1*	−2.0614							
*Dum2*		−2.8489						
*Dum3*			−5.4705 **					
*Dum30*				−3.0401				
*Dum40*					−6.3484			
*Nwom*						−3.681 ***		
*Pwom*							−36.5536 ***	
*Blau*								−23.0494 **
*IndepMem*	0.0266	0.0347	0.0404	0.0407	0.0368	0.0304	0.0386	0.0327
*AveTen*	2.7520 ***	2.7304 ***	2.6541 ***	2.8171 ***	2.7963 ***	2.581 ***	2.6246 ***	2.6624 ***
*EnvTra*	16.0769 ***	15.9772 ***	15.9190 ***	15.5274 ***	15.511 ***	15.2816 ***	15.0571 ***	15.3022 ***
*Ndir*	1.7321 **	1.7349 **	1.6590 **	1.9143 **	1.8881 **	2.2511 ***	1.9299 **	1.9259 **
*Temp*	0.2503	0.3873	0.4329	−0.1170	−0.0025	0.0704	−0.0773	0.0180
*ROA*	−0.0021	−0.1032	−0.0972	−0.1039	−0.1074	−0.0715	−0.0771	−0.0808
N	851	851	851	851	851	851	851	851
R^2^ (within)	0.5273	0.5281	0.5321	0.5280	0.5286	0.5371	0.5358	0.5333
*p* value (F)	0.0000	0.0000	0.0000	0.0000	0.0000	0.0000	0.0000	0.0000
*p* value (Hausman: FE/RE)	0.0000	0.0000	0.0000	0.0000	0.0000	0.0000	0.0000	0.0000
*p* value (Hausman: FEIV/FE)	0.9995	0.9995	0.9995	0.9944	0.9997	0.9629	0.9639	0.9912

Source: authors, based on Refinitiv [112]. Compiled using Stata 16, StataCorp LLC, Badajoz, Spain. *** significant at the 99% level, ** significant at the 95% level. R2 (within): coefficient of determination of the transformed model. *p* value (F): *p* value of the test of model significance. *p* value (Hausman: FE/RE): *p* value of the Hausman test under the null hypothesis of absence of correlation between the explanatory variables and the individual unobservable effect. *p* value (Hausman: FEIV/FE): *p* value of the Hausman test under the null hypothesis of absence of correlation between the explanatory variables and the error term. Time dummies are omitted for reasons of brevity and practicality. The estimation was performed with errors that are robust to heteroscedasticity and autocorrelation.

**Table 10:** Dependent variable: *InnoSc*. Fixed effects estimator (2002–2019).

Variables	(1)	(2)	(3)	(4)	(5)	(6)	(7)	(8)
*Dum1*	0.5448							
*Dum2*		−0.6150						
*Dum3*			−0.3320					
*Dum30*				6.1967 *				
*Dum40*					9.2036			
*Nwom*						2.5081 **		
*Pwom*							12.8849	
*Blau*								3.4029
*IndepMem*	−0.1794 **	−0.1799 *	−0.1801 *	−0.1905 **	−0.1816 **	−0.1799 **	−0.1804 **	−0.1789 *
*AveTen*	0.0820	0.0636	0.0668	0.0930	0.1182	0.2107	0.1467	0.1000
*EnvTra*	5.3199 *	5.2999 *	5.3114 *	5.7320 **	5.6523 **	5.8606 **	5.6013 **	5.4328 *
*Ndir*	0.7953	0.8299 *	0.8138 *	0.7862	0.8537	0.4121	0.8649	0.8789 *
*Temp*	−1.9699	−1.9323	−1.9534	−1.7054	−1.9651	−1.8544	−1.9879	−2.0265
*ROA*	0.0799 *	0.0831 *	0.0825 *	0.0724 *	0.0801 *	0.0561	0.0705	0.0775 *
N	851	851	851	851	851	851	851	851
R^2^ (within)	0.2400	0.2401	0.2400	0.2491	0.2474	0.2499	0.2428	0.2409
*p* value (F)	0.0000	0.0000	0.0000	0.0000	0.0000	0.0000	0.0000	0.0000
*p* value (Hausman: FE/RE)	0.4520	0.5692	0.4961	0.7653	0.6924	0.9599	0.8575	0.6886
*p* value (Hausman: FEIV/FE)	0.9999	0.9999	0.9999	0.9905	0.9999	0.9997	0.9999	0.9999

Source: authors, based on Refinitiv [112]. Compiled using Stata 16, StataCorp LLC, Badajoz, Spain. ** significant at the 95% level, * significant at the 90% level. R2 (within): coefficient of determination of the transformed model. *p* value (F): *p* value of the test of model significance. *p* value (Hausman: FE/RE): *p* value of the Hausman test under the null hypothesis of absence of correlation between the explanatory variables and the individual unobservable effect. *p* value (Hausman: FEIV/FE): *p* value of the Hausman test under the null hypothesis of absence of correlation between the explanatory variables and the error term. Time dummies are omitted for reasons of brevity and practicality. The estimation was performed with errors that are robust to heteroscedasticity and autocorrelation.

## Data Availability

The data presented in this study are available in the Thomson Reuters Eikon database [112].
